# Frontal-executive dysfunction affects dementia conversion in patients with amnestic mild cognitive impairment

**DOI:** 10.1038/s41598-020-57525-6

**Published:** 2020-01-21

**Authors:** Young Hee Jung, Seongbeom Park, Hyemin Jang, Soo Hyun Cho, Seung Joo Kim, Jun Pyo Kim, Sung Tae Kim, Duk L. Na, Sang Won Seo, Hee Jin Kim

**Affiliations:** 1 Department of Neurology, Myongji Hospital, Hanyang University, Goyang, Korea; 2 Department of Neurology, Samsung Medical Center, Sungkyunkwan University School of Medicine, Seoul, Korea; 3 Neuroscience Center, Samsung Medical Center, Seoul, Korea; 4 Samsung Alzheimer Research Center, Samsung Medical Center, Seoul, Korea; 5 Department of Health Sciences and Technology, SAIHST, Sungkyunkwan University, Seoul, Korea; 6 Stem cell & Regenerative Medicine Institute, Samsung Medical Center, Seoul, Korea; 7 Department of Clinical Research Design & Evaluation, SAIHST, Sungkyunkwan University, Seoul, Korea; 8 Department of Neurology, Chonnam National University Hospital, Chonnam National University Medical School, Gwangju, Korea; 9 Department of Neurology, Gyeongsang National University School of Medicine and Gyeongsang National University Changwon Hospital, Changwon, Korea; 10 Department of Radiology, Samsung Medical Center, Sungkyunkwan University School of Medicine, Seoul, Korea

**Keywords:** Alzheimer's disease, Alzheimer's disease

## Abstract

Among mild cognitive impairment (MCI) patients, those with memory impairment (amnestic MCI, aMCI) are at a high risk of dementia. However, the precise cognitive domain, beside memory, that predicts dementia conversion is unclear. Therefore, we investigated the cognitive domain that predicts dementia conversion in a longitudinal aMCI cohort. We collected data of 482 aMCI patients who underwent neuropsychological tests and magnetic resonance imaging at baseline and were followed for at least 1 year. The patients were categorized according to number (1–4) and type of impaired cognitive domains (memory, language, visuospatial, and frontal-executive function). We evaluated dementia conversion risk in each group when compared to single-domain aMCI after controlling for age, education, diabetes and dyslipidemia. Baseline cortical thickness of each group was compared to that of 410 cognitively normal controls (NCs) after controlling for age, intracranial volume, diabetes and dyslipidemia. Compared to single-domain aMCI, aMCI patients with frontal-executive dysfunction at baseline had a higher risk of dementia conversion than aMCI patients with visuospatial or language dysfunction. Compared to NCs, aMCI patients with frontal-executive dysfunction had overall cortical thinning including frontal areas. Our findings suggest that aMCI patients with frontal-executive dysfunction have poor prognosis and,thus, should be considered for intervention therapy with a higher priority among aMCI patients.

## Introduction

Mild cognitive impairment (MCI) is a transitional state between normal aging and dementia. MCI patients show dysfunction in one or more of the four major cognitive domains, namely language, visuospatial, memory, and frontal-executive function. Among the MCI patients, it has been well established that those with memory impairment (amnestic MCI, aMCI) are at a high risk of dementia, especially Alzheimer’s dementia (AD) type^1^. Additionally, previous studies have reported that among aMCI patients, multiple-domain cognitively impaired patients have poor prognosis compared to single-domain memory impaired patients^2^. However, which cognitive domain, beside memory, that predicts dementia or AD conversion is not well established^3^.

aMCI is a heterogenous group with varied phenotypes, pathologies, and prognoses. Many studies have attempted the to classify aMCI patients into homogenous groups using various tools^3,4^. Neuropsychological test is a widely-used, simple tool that can be easily utilized by primary physicians. Thus, although AD biomarkers such as amyloid and tau can be assessed via cerebrospinal fluid (CSF) or positron emission tomography (PET) scan in referral hospitals, predicting the prognosis of aMCI patients through neuropsychological test holds greater importance in clinical practice.

Therefore, we aimed to identify the cognitive domain that predicts dementia or AD conversion among aMCI patients. We categorized the aMCI patients according to the number (1–4) and combination of impaired cognitive domains (memory, language, visuospatial, and frontal-executive function). We investigated the combination of impaired cognitive domain that shows poor prognosis in a longitudinal aMCI cohort. Furthermore, we investigated whether the baseline cortical thickness differed according to the combination of the impaired cognitive domain.

## Results

### Baseline characteristics of participants

Compared to NC, total aMCI patients showed higher prevalence of diabetes (p = 0.001) and dyslipidaemia (p = 0.006) and higher proportion of APOE4 carriers (p < 0.001). Compared to 1M-MCI patients, 3MLF-MCI patients were older (p = 0.001). Compared to 1M-MCI patients, 2MV-MCI (p = 0.003) and 4MLVF-MCI (p < 0.001) patients were less educated. Compared to 1M-MCI patients, 3-domain MCI (p = 0.020) patients had lower proportions of APOE 4 carriers. Compared to 2MV-MCI patients, 2ML-MCI patients were older (p = 0.001). Compared to 3MLF-MCI patients, 3MVF-MCI patients were younger (p = 0.007) and less educated (p = 0.001) (Table 1).

**Table 1:** Baseline characteristics of participants.

	NC	Total aMCI	1-domain	2-domain MCI				3-domain MCI				4-domain MCI
			1M-MCI	2-domains, total	2ML-MCI	2MV-MCI	2MF-MCI	3-domains, total	3MLV-MCI	3MLF-MCI	3MVF-MCI	4MLVF-MCI
**N**	410	482	157	166	59	33	74	115	23	58	34	44
**Age**	72.2 ± 5.3	71.6 ± 7.8	69.9 ± 8.1	71.6 ± 7.7	75.4 ± 6.7^b^	67.9 ± 8.5^c^	70.3 ± 6.7^c^	73.2 ± 7.2^b^	73.3 ± 7.3	74.9 ± 6.5^b^	70.2 ± 7.5^d^	72.9 ± 7.5
**Female, n (%)**	279 (68.0)	288 (59.8)^a^	95 (60.5)	98 (59.0)	36 (61.0)	22 (66.7)	40 (54.1)	65 (56.5)	16 (69.6)	31 (53.4)	18 (52.9)	30 (68.2)
**Education**	10.3 ± 5.3	10.8 ± 5.1	11.7 ± 5.0	10.7 ± 5.1	12.1 ± 5.0	8.3 ± 4.1^b^	10.7 ± 5.2	10.2 ± 5.1	10.3 ± 4.8	11.6 ± 4.8	7.6 ± 4.9^b,d^	9.2 ± 5.5^b^
**MMSE**	27.9 ± 2.4	25.5 ± 3.1^a^	26.4 ± 3.0	25.8 ± 2.9	26.0 ± 3.0	26.1 ± 2.6	25.6 ± 3.1	24.7 ± 2.7^b^	25.0 ± 2.5^b^	25.0 ± 2.7^b^	23.9 ± 2.9^b,^	23.6 ± 3.5^b^
**Vascular Risk Factors**												
Diabetes, n (%)	93/402 (23.1)	153 (31.7)^a^	45/156 (28.8)	53 (31.9)	17 (28.8)	15 (45.5)	21 (28.4)	34 (29.6)	4 (17.4)	16 (27.6)	14 (41.2)	21/43 (48.8) ^b^
Hypertension, n (%)	170 (41.5)	213 (44.2)	45 (47.1)	74 (44.6)	22 (37.3)	12 (36.4)	40 (54.1)	46 (40.0)	7 (30.4)	24 (41.4)	14 (44.1)	19 (43.2)
Dyslipidemia, n (%)	154 (37.6)	139 (28.8)^a^	44 (28.0)	50 (30.1)	23 (39.0)	7 (21.2)	20 (27.0)	28 (24.3)	5 (21.7)	16 (27.6)	7 (20.6)	17 (38.6)
**APOE4 Carrier, n (%)**	86 (21.0)	160/430 (38.7)^a^	58/132 (43.9)	61/145 (42.1)	23/53 (43.4)	11/28 (39.3)	27/64 (42.2)	28/97 (28.9)^b^	3/15 (20.0)	17/54 (31.5)	8/28 (28.6)	13/39 (33.3)
**Follow-up Duration, Months**		44.5 ± 20.6	44.8 ± 20.6	44.2 ± 21.0	44.5 ± 20.7	47.1 ± 22.3	42.7 ± 20.7	43.2 ± 20.7	43.4 ± 18.4	43.8 ± 20.0	41.9 ± 23.5	47.4 ± 19.3

^a^p < 0.05 Comparison between NC and total aMCI.

^b^p < 0.05 Comparison between 1M-MCI and each MCI subtype.

^c^p < 0.05 Comparison between 2ML-MCI and 2MV-MCI or between 2ML-MCI and 2MF-MCI.

^d^p < 0.05 Comparison between 3MLF-MCI and 3MVF-MCI.

1M-MCI = single amnestic mild cognitive impairment; 2ML-MCI = amnestic MCI with memory and language dysfunction; 2MV-MCI = amnestic MCI with memory and visuospatial dysfuction; 2MF-MCI = amnestic MCI with memory and frontal-executive dysfunction; 3MLV-MCI = amnestic MCI with memory, language, and visuospatial dysfunction; 3MLF-MCI = amnestic MCI with memory, language, and frontal-executive dysfunction; 3MVF-MCI = amnestic MCI with memory, visuospatial, and frontal-executive dysfunction; 4MLVF-MCI = amnestic MCI with memory, language, visuospatial, and frontal-executive dysfunction;

MMSE: Mini-Mental Status Examination; APOE4: Apolipoprotein E e4.

### Dementia conversion risk according to baseline cognitive profile

Among 482 aMCI patients, 182 (37.8%) converted to dementia and most of them (164 patients) were of the AD type. In 1M-MCI subgroup, 28.7% of patients progressed to dementia, (26.8% were of AD type and 1.9% were of other dementia types). In the 2-domain MCI subgroup, 38.6% of patients progressed to dementia (35.5% were of AD type and 3.0% were of other dementia types). In the 3-domain MCI subgroups 43.5% of patients progressed to dementia (40.9% were of AD type and 2.6% were of other dementia types). In the 4-domain MCI subgroup, 52.3% of patients progressed to dementia (36.4% were of AD type and 15.9% were of other dementia types) (Table 2).

**Table 2:** Dementia conversion according to subgroups.

	total aMCI	1-domain	2-domain MCI				3-domain MCI				4-domain MCI
		1M-MCI	2 domains, total	2ML-MCI	2MV-MCI	2MF-MCI	3 domains, total	3MLV-MCI	3MLF-MCI	3MVF-MCI	4MLVF-MCI
N	482	157	166	59	33	74	115	23	58	34	44
Reverse to Normal, n (%)	16 (3.3)	11 (7.0)	4 (2.4)	1 (1.7)	0 (0)	3 (4.1)	1/115 (0.9)^a^	0 (0)	1 (1.7)	0 (0)	0 (0)
Dementia Conversion, n (%)	182 (37.8)	45 (28.7)	64 (38.6)	19 (32.2)	11 (33.3)	34 (45.9)^a^	50 (43.5)^a^	8 (34.8)	28 (48.3)^a^	14 (41.2)	23 (52.3)^a^
Conversion to Alzheimer’s dementia, n (%)	164 (34.0)	42 (26.8)	59 (35.5)	18 (30.5)	10 (30.3)	31 (41.9)^a^	47 (40.9)^a^	8 (34.8)	26 (44.8)^a^	13 (38.2)	16 (36.4)^a^
Conversion to Other Dementia, n (%)	18 (3.7)	3 (1.9) [VD 2, svPPA 1]	5 (3.0) [VD 4, PSP 1]	1 (1.7) [VD 1]	1 (3.0) [VD 1]	3 (4.1)^a^ [VD 2, PSP 1]	3 (2.6)^b^ [NPH 1, PSP 1, DLB 1]	0 (0)	2 (3.4)^a^ [NPH 1, PSP 1]	1 (2.9) [DLB 1]	7 (15.9)^a^ [VD 3, PSP 2, DLB 1, Mixed dementia 1]

^a^p < 0.05 Comparison between 1M-MCI and each MCI subtype

1M-MCI = single amnestic mild cognitive impairment; 2ML-MCI = amnestic MCI with memory and language dysfunction; 2MV-MCI = amnestic MCI with memory and visuospatial dysfuction; 2MF-MCI = amnestic MCI with memory and frontal-executive dysfunction; 3MLV-MCI = amnestic MCI with memory, language, and visuospatial dysfunction; 3MLF-MCI = amnestic MCI with memory, language, and frontal-executive dysfunction; 3MVF-MCI = amnestic MCI with memory, visuospatial, and frontal-executive dysfunction; 4MLVF-MCI = amnestic MCI with memory, language, visuospatial, and frontal-executive dysfunction

VD = vascular dementia; PSP = progressive supranuclear palsy; DLB = dementia with Lewy body; NPH = normal pressure hydrocephalus, svPPA = semantic variant primary progressive aphasia.

**Figure 1:**
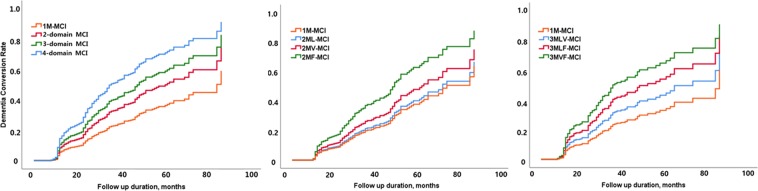
Cumulative dementia conversion rate in each cognitive profile. 1M-MCI = single amnestic mild cognitive impairment; 2ML-MCI = amnestic MCI with memory and language dysfunction; 2MV-MCI = amnestic MCI with memory and visuospatial dysfuction; 2MF-MCI = amnestic MCI with memory and frontal-executive dysfunction; 3MLV-MCI = amnestic MCI with memory, language, and visuospatial dysfunction; 3MLF-MCI = amnestic MCI with memory, language, and frontal-executive dysfunction; 3MVF-MCI = amnestic MCI with memory, visuospatial, and frontal-executive dysfunction; 4MLVF-MCI = amnestic MCI with memory, language, visuospatial, and frontal-executive dysfunction.

Table 3 and Fig. 1 show dementia conversion risk of each cognitive profile compared to 1M-MCI patients. Compared to 1M-MCI patients, 2-domain (HR = 1.59, P = 0.019), 3-domain (HR = 2.06, P = 0.001), and 4-domain aMCI patients (HR = 2.93, P < 0.001) were at higher risk of dementia conversion. When we further analysed each cognitive profile, aMCI patients with frontal-executive dysfunction (2MF-MCI [HR = 2.02, P = 0.002], 3MLF-MCI [HR = 2.22, P = 0.001], 3MVF-MCI [HR = 2.75, P = 0.002], and 4MLVF-MCI [HR = 2.93, P < 0.001]) were at higher risk of dementia conversion. The results did not change when we analysed the risk of AD conversion.

**Table 3:** Hazard ratios of dementia and Alzheimer’s dementia (AD) conversion according to cognitive profile.

	Hazard ratio of demnetia conversion (CI 0.95)	P value	Hazard ratio of AD conversion (CI 0.95)	P value
1M-MCI	REF		REF	
2-domain MCI	1.59 (1.08–2.33)	0.019	1.57 (1.05–2.35)	0.027
2MV-MCI	1.17 (0.60–2.26)	0.650	1.13 (0.57–2.26)	0.726
2ML-MCI	1.44 (0.84–2.46)	0.188	1.46 (0.84–2.54)	0.182
2MF-MCI	2.02 (1.29–3.16)	0.002	1.97 (1.23–3.14)	0.005
3-domain MCI	2.06 (1.36–3.13)	0.001	2.08 (1.35–3.21)	0.001
3MLV-MCI	1.74 (0.81–3.72)	0.156	1.88 (0.87–4.06)	0.106
3MLF-MCI	2.22 (1.38–3.57)	0.001	2.22 (1.35–3.63)	0.002
3MVF-MCI	2.75 (1.47–5.16)	0.002	2.76 (1.44–5.31)	0.002
4-domain MCI	2.93 (1.74–4.94)	0.000	2.19 (1.20–3.96)	0.010

Cox proportional hazard ratio of each aMCI subgroup was analyzed, compared to that of 1M-MCI group after controlling age, education year, diabetes and dyslipidemia. (confidence interval = 0.95).

CI = confidence interval; 1M-MCI = single amnestic mild cognitive impairment; 2ML-MCI = amnestic MCI with memory and language dysfunction; 2MV-MCI = amnestic MCI with memory and visuospatial dysfuction; 2MF-MCI = amnestic MCI with memory and frontal-executive dysfunction; 3MLV-MCI = amnestic MCI with memory, language, and visuospatial dysfunction; 3MLF-MCI = amnestic MCI with memory, language, and frontal-executive dysfunction; 3MVF-MCI = amnestic MCI with memory, visuospatial, and frontal-executive dysfunction; 4MLVF-MCI = amnestic MCI with memory, language, visuospatial, and frontal-executive dysfunction.

### Baseline cortical thickness in each cognitive profile

**Figure 2:**
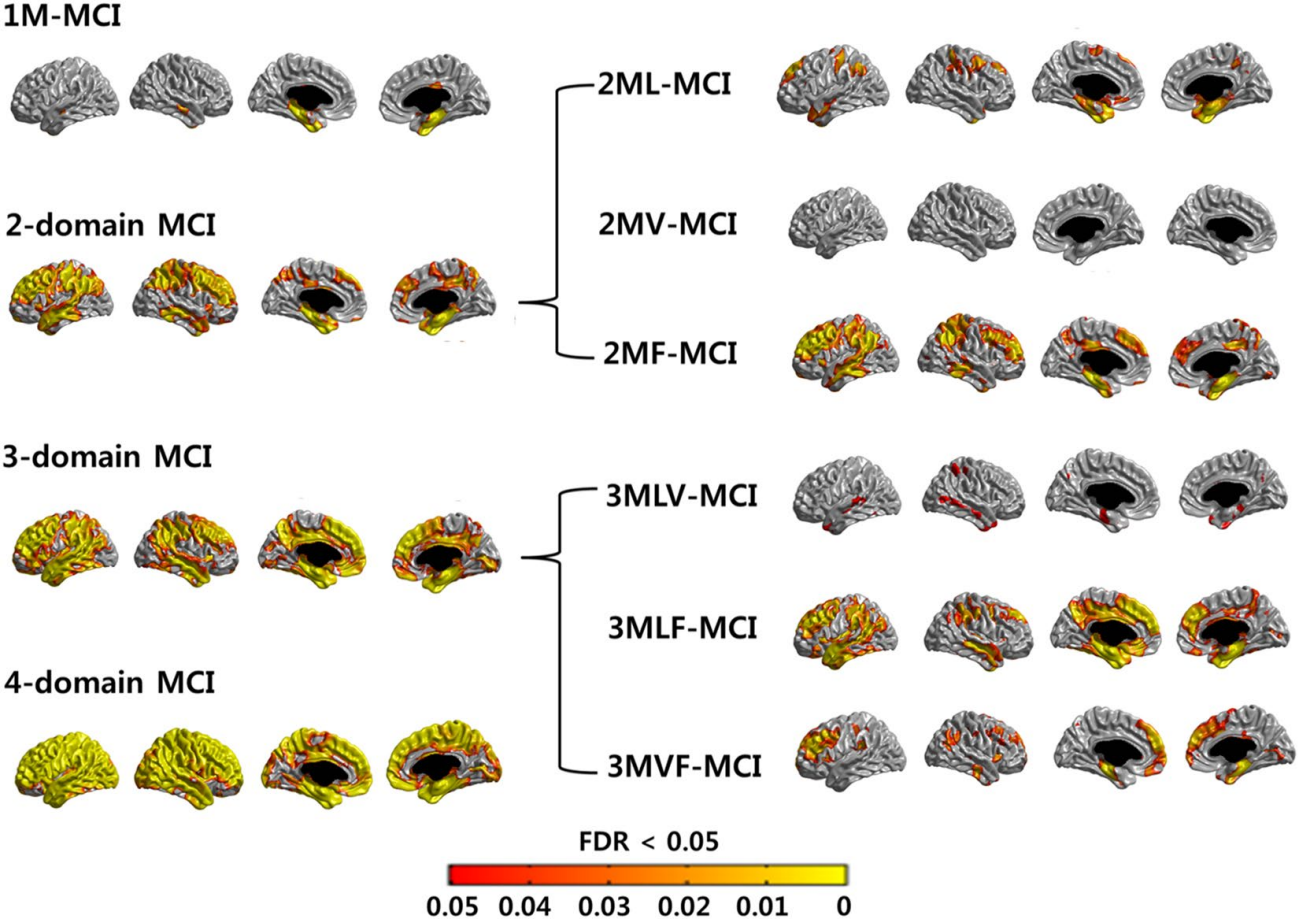
Baseline cortical thinning in aMCI patients of each cognitive profile. Cortical thickness of each group was compared to that of cognitive normal controls after controlling for age,intracranial volume, diabetes and dyslipidemia. (false discovery rate [FDR] corrected p < 0.05). 1M-MCI = single amnestic mild cognitive impairment; 2ML-MCI = amnestic MCI with memory and language dysfunction; 2MV-MCI = amnestic MCI with memory and visuospatial dysfuction; 2MF-MCI = amnestic MCI with memory and frontal-executive dysfunction; 3MLV-MCI = amnestic MCI with memory, language, and visuospatial dysfunction; 3MLF-MCI = amnestic MCI with memory, language, and frontal-executive dysfunction; 3MVF-MCI = amnestic MCI with memory, visuospatial, and frontal-executive dysfunction; 4MLVF-MCI = amnestic MCI with memory, language, visuospatial, and frontal-executive dysfunction.

Cortical thickness of patients in each cognitive profile was compared to cortical thickness of NCs (Fig. 2). 1M-MCI patients showed cortical thinning in the bilateral medial temporal areas, while 2-domain MCI patients showed cortical thinning in more extended areas involving the bilateral frontal, parietal, and temporal areas. The 3-domain MCI patients showed cortical thinning in more extended areas sparing the primary motor, sensory, and visual cortices. The 4-domain aMCI patients showed cortical thinning in almost the entire cortex, sparing the primary visual cortex.

Among the 2-domain MCI patients, 2MV-MCI did not show significant cortical thinning, while those with language dysfunction showed bilateral medial temporal atrophy. 2MF-MCI showed cortical thinning in more extended areas including frontal and parietal areas.

Among the 3-domain MCI patients, 3MVL-MCI did not show significant cortical thinning, while 3MVF-MCI showed bilateral frontal cortical thinning. Furthermore, 3MLF-MCI showed cortical atrophy in more extended areas, including bilateral frontal, temporal, and parietal areas.

### Comparison of baseline cortical thickness and neuropsychological profiles between dementia non-converters and dementia converters

We compared the cortical thickness between dementia converters and non-converters. The dementia converters showed thinner cortices in the frontal areas as well as temporal and parietal areas than non-converters at baseline (eFig. 1). Likewise, the dementia converters showed more severe cognitive impairment in all cognitive domains including memory, language, visuospatial, and frontal-executive functions compared to non-converters at baseline (eTable 1).

## Discussion

We investigated the cognitive domain that affects dementia conversion risk in a large longitudinal aMCI cohort. We found that among aMCI patients, those with additional frontal-executive dysfunction rather than language or visuospatial dysfunction were at a higher risk of dementia conversion compared with single-domain aMCI patients. We also found that aMCI patients with frontal-executive dysfunction showed diffuse cortical thinning, especially in the frontal areas. Our findings suggest that the presence of frontal-executive dysfunction in addition to memory impairment is a poor prognostic sign, and thus, patients with such cognitive profiles should be considered for intervention therapy with a higher priority among aMCI patients.

We found that frontal-executive dysfunction in aMCI increased the risk of dementia conversion. The results were consistent among 2-domain aMCI patients and 3-domain aMCI patients. Previous studies suggested several cognitive profiles that were associated with poor prognosis in aMCI patients: multiple domain aMCI, late aMCI^5^, or both memory (visual and verbal) impairments^6^. However, a few studies evaluated the prognostic value of each cognitive domain. Our study showed that compared to 1M-MCI, aMCI patients with frontal-executive dysfunction (2MF-MCI, 3MLF-MCI, 3MVF-MCI, and 4MLVF-MCI) were at higher risk of dementia conversion, while aMCI patients with visuospatial or language dysfunction (2ML-MCI, 2MV-MCI, and 3MLV-MCI) were not. Our results add on the existing knowledge regarding aMCI prognosis.

Our study showed that frontal-executive dysfunction, but not language or visuospatial dysfunction, was a poor prognostic marker in aMCI patients. Previous results about cognitive domains, other than memory, that predict dementia conversion in aMCI patients have been controversial. Some studies reported language dysfunction as a poor prognostic factor^7,8^, while other studies suggested frontal-executive dysfunction as a poor prognostic factor^9^. Recent studies suggested mild behaviour impairment as one of the poor prognostic factors^10,11^.

When aMCI patients with frontal-executive dysfunction developed dementia, it was mostly the AD type rather than FTD. Among 74 2MF-MCI patients, 34 developed dementia, which was of AD type in 31 (91.2%) patients. The proportion of AD type dementia conversion was similar to a previous study from Fisher *et al*., who reported that 91.6% of patients who converted to dementia were of AD type^12^.

Among 58 MLF-MCI patients, 28 developed dementia, which was of AD type in 26 (92.9%) patients. Among 34 3MVF patients, 14 developed dementia, which was of AD type in 13 (92.9%) patients. This might suggest that additional frontal executive function in aMCI increases risk for AD rather than FTD. However, since frontal-executive dysfunction is a hallmark of FTD symptomatology and FTD may have a sneaky onset^13^, it is possible that these patients may develop FTD.

We found that aMCI patients with frontal-executive dysfunction had more severe baseline cortical atrophy, especially in the frontal areas. As cortical atrophy is a marker of neurodegeneration^14,15^, our results suggest that presence of frontal-executive dysfunction is related to more severe neurodegeneration. It is well known that medial temporal atrophy is a poor prognostic marker in aMCI^16^. Our results add to the previous knowledge that extended cortical atrophy, especially to the frontal area, as well as medial temporal atrophy, are poor prognostic markers in aMCI patients.

Our data also showed that dementia converters had diffuse cortical thinning and cognitive impairment in all cognitive domains compared to dementia non-converters at baseline. These findings may provide additional evidence that AD pathology does not involve only the temporal area but spreads also to the frontal regions^17–19^.

There are several limitations in our study. First, we diagnosed patients based on the findings of neuropsychological tests and brain MRI and did not measure AD biomarkers. Second, this study was based on data from a single centre. To confirm our findings, a multi-centre study with a longer follow-up period is necessary.

Our study is noteworthy in that the results enable prediction of the prognosis of aMCI patients according to the cognitive profile. aMCI is a heterogenous group with various phenotypes and various underlying pathologies. Classifying and predicting the prognosis of aMCI patients according to the results of neuropsychological tests is the first step towards precision medicine. Our results may help physicians decide whether to further evaluate aMCI patients using brain imaging or AD biomarkers. We suggest that aMCI patients with frontal-executive dysfunction should be considered for high-priority intervention therapy to delay cognitive decline.

## Methods

### Participants

We consecutively collected data of 498 aMCI patients from the Memory Clinic at Samsung Medical Center in Seoul, South Korea. The patients underwent detailed neuropsychological testing and brain magnetic resosnance imaging (MRI) from July 2007 to December 2012 and were followed-up for more than 12 months. All patients were in accordance with Petersen’s clinical criteria for MCI^20^ with the following modifications: (1) subjective memory problems reported by the patient or caregiver, (2) normal activities of daily living (ADL) as judged by an interview with a clinician and Seoul–Instrumental ADL test (with a score < 8)^21^, (3) objective memory decline below the 16^th^ percentile or the norm determined by neuropsychological tests, and (4) no dementia. The exclusion criteria included a history of traumatic brain injury, cortical stroke, seizure, brain surgery, and existing systemic diseases with potential cognitive effects, and severe white matter hyperintensities (WMH) which was defined as deep WMH ≥ 25 mm and periventricular WMH ≥ 10 mm. We also excluded patients who met the Diagnostic and Statistical Manual of Mental Disorders (Fourth Edition) criteria for psychotic or mood disorders, such as schizophrenia or major depressive disorder^22^.

Of the 498 patients who met the aforementioned inclusion and exclusion criteria, we excluded 16 patients due to error during cortical thickness analysis. Therefore, the final sample size included in the anlysis consisted of 482 aMCI patients.

We categorized all aMCI patients according to the number (1–4) and combination of impaired cognitive domains (memory, language, visuospatial, and frontal-executive function) based on neuropsychological tests. Those who only had memory impairment were classified as 1M-MCI. Those who had two domains impaired were categorized into three groups: 2ML-MCI (memory and language dysfunction), 2MV-MCI (memory and visuospatial dysfunction), and 2MF-MCI (memory and frontal-executive dysfunction). Furthermore, those who had three domains impaired were categorized into three groups: 3MLV-MCI (memory, language, and visuospatial dysfunction), 3MLF-MCI (memory, language, and frontal-executive dysfunction), and 3MVF-MCI (memory, visuospatial, and frontal-executive dysfunction). Patients who had four domains impaired were classified as 4MLVF-MCI (memory, language, visuospatial, and frontal-executive dysfunction).

We also recruited 410 age-matched normal controls (NCs) using the following criteria: (1) no history of neurologic or psychiatric disorders, (2) normal cognitive function determined using neuropsychological tests, and (3) normal activities of daily living as determined using the Seoul–Instrumental ADL test (with a score of < 8)^21^.

This study was approved by the Institutional Review Board of Samsung Medical Center and the methods were carried out in accordance with the approved guidelines. This manuscript does not contain information or image that can lead to identification of a study participant. The requirement for participant’s consent was waived by the Institutional Review Board of Samsung Medical Center since we used retrospective de-identified data collected during health exam visits.

### Neuropsychological tests

All patients underwent the Seoul Neuropsychological Screening Battery^23^, which contains tests for language, visuospatial function, verbal and visual memory, and frontal-executive function. Language was assessed as abnormal when the score on the Korean version of the Boston Naming Test^24^ was below the 16^th^ percentile of the norm. Visuospatial function was considered abnormal when the copying score of the Rey-Osterrieth Complex Figure Test (RCFT) was below the 16^th^ percentile of the norm; memory function was considered abnormal when the score of 20-min delayed recall on Seoul Verbal Learning Test and RCFT was below the 16^th^ percentile of the norm. Frontal-executive function was examined in three parts: motor executive function (contrasting program, Go/No-go, fist-edge-palm, alternating hand movement, alternative square and triangle, and Luria loop), Controlled Oral Word Association Test (COWAT), and Stroop Test. Frontal-executive function was considered abnormal when at least two of the three parts were below the 16^th^ percentile of the norm. The reference score for each of the aforementioned tests was obtained from the assessment of 447 normal Korean participants. General cognition was assessed by the Korean version of the Mini-Mental State Examination.

### Longitudinal follow-up of aMCI patients

All patients were followed up for more than 12 months (mean 44.5 months). The point of dementia conversion was determined by neurologists (DL Na, SW Seo, and HJ Kim) based on clinical interviews and neuropsychological tests, including the MMSE and the instrumental ADL scale. For patients who did not take detailed neuropsychological tests (n = 5), neurologists determined dementia conversion based on clinical interviews and the MMSE, the Geriatric Deterioration Scale^25^. Dementia was defined as cognitive impairment that affects several domains and/or neurobehavioral symptoms, resulting in functional impact on daily life^26^. Then, the type of dementia was assessed according to each clinical diagnostic criteria. AD was clinically defined according to the clinical diagnostic criteria of 2011 National Institute on Aging-Alzheimer’s Association workgroups on diagnostic guidelines for Alzheimer’s disease^27^. Progressive supranuclear palsy (PSP) was identified according to the NINDS-SPSP clinical diagnostic criteria, published in 1996. Dementia with Lewy body (DLB) was defined according to the diagnostic criteria 4^th^ report of DLB consortium^28^. In addition, frontotemporal dementia (FTD) was defined according to the clinical criteria of the report of the work group of FTD and Pick’s disease^29^.

### Acquisition of MRI and cortical thickness measurements

An Achieva 3.0 Tesla MRI scanner (Philips, Best, The Netherlands) was used to acquire 3D T1 turbo field echo (TFE) MRI data from all participants using the following imaging parameters: sagittal slice thickness, 1.0 mm; over contiguous slices with 50% overlap; no gap; repetition time, 9.9 ms; echo time, 4.6 ms; flip angle, 8 degrees; and matrix size of 240 × 240 pixels reconstructed to 480 × 480 over a field of view of 240 mm.

Images were processed using the standard Montreal Neurological Institute anatomic pipeline. The native MRI images were registered into a standardized stereotaxic space using linear transformation^30^. The N3 algorithm was used to correct images for intensity nonuniformities resulting from inhomogeneities in the magnetic field^31^. The registered and corrected volumes were classified into white matter, gray matter, CSF, and background using a 3D stereotaxic brain mask and the Intensity-Normalized Stereotaxic Environment for Classification of Tissues algorithm^32^. The surfaces of the inner and outer cortices were automatically extracted using the Constrained Laplacian-based Automated Segmentation with Proximities algorithm^33^.

Cortical thickness was calculated as the Euclidean distance between the linked vertices of the inner and outer surfaces, after the application of an inverse transformation matrix to cortical surfaces and reconstructing them in the native space^33^. To control for brain size, we computed the intracranial volume (ICV) using classified tissue information and a skull mask, which was acquired from the T1-weighted image^34^. ICV is defined as the total volume of gray matter, white matter, and CSF with consideration to voxel dimension. Classified gray matter, white matter, CSF, and background within the mask were transformed back into the individual native space.

### Statistical analyses

To evaluate the differences in demographics, we performed analysis of variance followed by Bonferroni’s post hoc analysis for continuous variables and Chi-squared test followed by Bonferroni’s post hoc analysis for categorical variables.

To assess whether the dementia risk differed according to the baseline cognitive profile, we applied Cox’s proportional hazards modelafter controlling for age, education level, diabetes, and dyslipidemia. The hazard ratio of each aMCI subgroup was compared to that of 1M-MCI group (confidence interval = 0.95).

We used MatLab (Version 2014b, Mathworks, Natick, USA) for MRI analyses. In order to compare cortical thickness topography, we used the Surfstat package created by Dr. Keith Worsley (http://www.math.mcgill.ca/keith/surfstat). We also compared localized cortical thickness between dementia converters and non-converters after controlling for age, ICV, diabetes, and dyslipidemia. The cortical surface model contained 81,924 vertices. We assessed statistical significance of difference between NCs and each aMCI subgroup for an individual vertex after correcting for multiple comparisons (false discovery rate at p-value < 0.05).

To compare neuropsychological profile between non-converters and converters, we used z-scores of each test, which were based on the mean and standard deviation of each measure in the age- and education- matched norms of 1,067 normal Korean participants. We used analysis of of covariance (ANCOVA) after controlling for diabetes and dyslipidemia.

A two-sided p-value < 0.05 was considered statistically significant. All analyses were performed using Predictive Analysis of Software Statistics Statistics 22 software (SPSS Inc., Chicago, IL, USA).

## Supplementary information

Supplementary information.
