# Supplementary material for: Frontal-executive dysfunction affects dementia conversion in patients with amnestic mild cognitive impairment
Source: Sci Rep. 2020 Jan 21;10:772. doi: 10.1038/s41598-020-57525-6 (PMC6972894; doi:10.1038/s41598-020-57525-6)
Supplement: Supplementary file 1 — Supplementary information. [file 41598_2020_57525_MOESM1_ESM.docx]

**Frontal-executive dysfunction affects dementia conversion in patients with amnestic mild cognitive impairment**

**Young Hee Jung^1,2,3^, Seongbeom Park^2,3,4^, Hyemin Jang^2,3,4^, Soo Hyun Cho^2,3,8^, Seung Joo Kim^2,3,9^, Jun Pyo Kim^2,3,4^, Sung Tae Kim^10^, Duk L. Na^2,3,4,5,6,7^, Sang Won Seo^2,3,4,7^, Hee Jin Kim^2,3,4*^**

^1^Department of Neurology, Myongji Hospital, Hanyang University, Goyang, Korea.

^2^Department of Neurology, Samsung Medical Center, Sungkyunkwan University School of Medicine, Seoul, Korea

^3^Neuroscience Center, Samsung Medical Center, Seoul, Korea

^4^Samsung Alzheimer Research Center, Samsung Medical Center, Seoul, Korea

^5^Department of Health Sciences and Technology, SAIHST, Sungkyunkwan University, Seoul, Korea

^6^Stem cell & Regenerative Medicine Institute, Samsung Medical Center, Seoul, Korea

^7^Department of Clinical Research Design & Evaluation, SAIHST, Sungkyunkwan University, Seoul, Korea

^8^Department of Neurology, Chonnam National University Hospital, Chonnam National University Medical School, Gwangju, Korea

^9^Department of Neurology, Gyeongsang National University School of Medicine and Gyeongsang National University Changwon Hospital, Changwon, Korea

^10^Department of Radiology, Samsung Medical Center, Sungkyunkwan University School of Medicine, Seoul, Korea

**^*^Corresponding author:** Hee Jin Kim, MD, PhD

Department of Neurology, Sungkyunkwan University School of Medicine, Samsung Medical Center, 50 Ilwon-dong, Gangnam-gu, Seoul 135-710, Republic of Korea

Tel.: +82-2-3410-6147

Fax: +82-2-3410-0052

E-mail: [evekhj@gmail.com](mailto:evekhj@gmail.com)

**
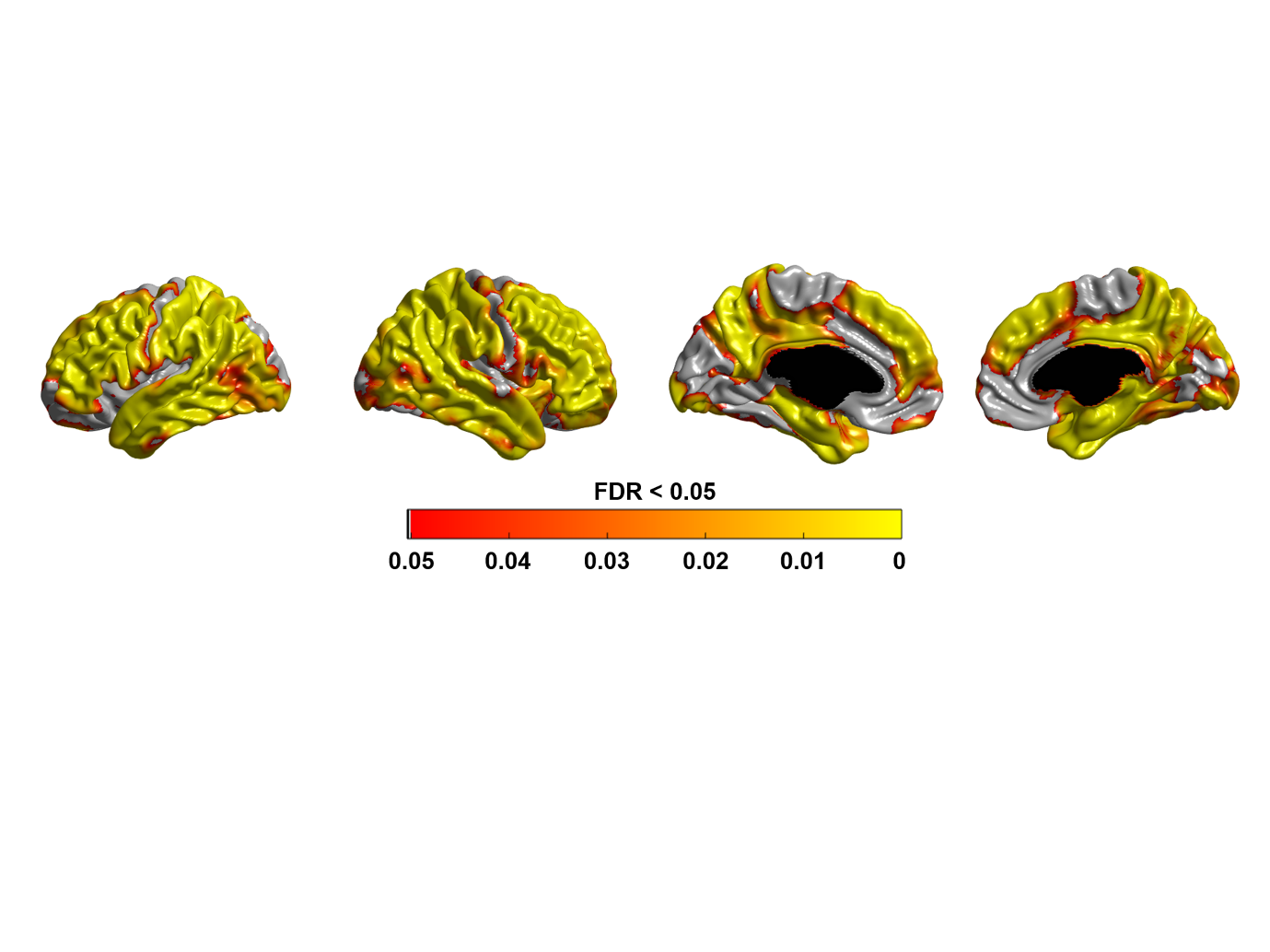
eFigure 1. Baseline cortical thickness of dementia converters compared with non-converters**

Cortical thickness of aMCI patients who converted to dementia was compared to that of non-converters after controlling for age, diabetes, dyslipidemia and intracranial volume. (false discovery rate [FDR] correction at p-value < 0.05 )

**eTable 1. Comparison of neuropsychological profile between dementia non-converters and dementia converters**

| **Neuropsychological test** | **Dementia Non-converter**  n = 300 | **Dementia Converter** n = 182 | **P value** |
| --- | --- | --- | --- |
| **Language** |  |  |  |
| K-BNT | -0.66 ± 1.31 | -1.16 ± 1.69 | < 0.001 |
| **Visuospatial** |  |  |  |
| RCFT copy | -0.42 ± 0.10 | -0.76 ± 0.13 | 0.037 |
| **Memory** |  |  |  |
| SVLT delayed recall | -1.40 ± 1.45 | -2.05 ± 1.38 | < 0.001 |
| RCFT delayed recall | -1.20 ± 0.88 | -1.79 ± 0.75 | < 0.001 |
| **Frontal** |  |  |  |
| COWAT, phonemic | -0.16 ± 0.07 | -0.42 ± 0.09 | 0.018 |
| Stroop, color reading | -0.67 ± 4.46 | -1.27 ± 1.42 | 0.087 |

Values are mean z-scores (mean ± standard deviation) of neuropsychological tests. Analysis of covariance (ANCOVA) was performed after controlling for diabetes and dyslipidemia

K-BNT = Korean version of Boston naming test; RCFT = Rey complex figure test; SVLT = Seoul verbal learning test; COWAT = controlled oral word association
